# Facilitating and inhibiting factors related to treatment adherence in women with polycystic ovary syndrome: A qualitative study

**Published:** 2017-09

**Authors:** Fatemeh Bazarganipour, Seyed Abdolvahab Taghavi, Helen Allan, Nazafarin Hosseini

**Affiliations:** 1 *Social Determinants of Health Research Center, Yasuj University of Medical Sciences, Yasuj, Iran.*; 2 *Fertility and Infertility Research Center, Hormozgan University of Medical Sciences, Bandar Abbas, Iran. *; 3 *Centre for Critical Research in Nursing and Midwifery, School of Health and Education, Middlesex University, London, England.*

**Keywords:** Polycystic ovarian syndrome, Adherence, Qualitative research

## Abstract

**Background:**

Adherence issues in polycystic ovary syndrome (PCOS) patients have not been examined thoroughly. Patients report prolonged periods of treatment and side effects of the drug as the most common reason for withdrawal from treatment. To improve the effective management of PCOS patients, it is fundamental to understand facilitating and inhibiting factors to treatment adherence.

**Objective:**

To explore facilitating/inhibiting factors related to treatment adherence among PCOS patients.

**Materials and Methods:**

This was a qualitative study with a purposive sample of women with confirmed diagnosis of PCOS. The data were collected via 20 in-depth semi-structured interviews with women aged between 21-34 yr. A qualitative content analysis was used to analyze the data.

**Results:**

Five themes were identified which described different types of facilitating/ inhibiting factors to treatment adherence. Inhibiting factors included financial issues, patient-related, disease-related, and health care provider-related factors; while social factors were found to be both facilitating and inhibiting.

**Conclusion:**

The findings suggest that successful adherence to PCOS treatment is highly dependent on patients recognizing and adapting to financial, social, and health care related inhibiting factors. It is also crucial for clinicians and policy makers to recognize these key inhibiting factors in order to improve treatment outcomes**.**

## Introduction

Polycystic ovary syndrome (PCOS) is the most common endocrine disorder in women of childbearing age. It is estimated that 16.5% of women suffer from the disease (1). The symptoms typically associated with PCOS include amenorrhea, oligomenorrhea, hirsutism, obesity, infertility, anovulation, and acne. These symptoms can lead to depression, marital and social maladjustment and impaired sexual functioning (2).

Effective treatment for PCOS may include oral contraceptives, progesterone, metformin, anti-androgens as well as lifestyle modification and/or weight loss (3). Therefore, achieving effective management of PCOS and reduction of clinical complications requires a prolonged compliance with multiple prescribed drugs and changes in lifestyle. The term compliance has mostly been superseded by the term adherence, a similar concept but one that has more positive implications for the physician/patient relationship. Using compliance was heavily criticized, as it conveyed a negative image of the relationship between patient and prescriber, where the role of the prescriber seemed to be to issue instructions to the patient who had to follow the prescriber’s orders. Noncompliance, then, was interpreted as patient incompetence – not being able to follow instructions, or deliberate, self-sabotaging behavior. The term adherence recognizes a patient’s right to choose to follow a doctor’s suggested treatment plan.

A World Health Organization report suggests that 50% of patients from developed countries with chronic disease do not use their medications as recommended (1). In developing countries, in addition to poor access to health care, lack of appropriate diagnosis and limited access to medicines, poor adherence seriously threatens any effort to tackle chronic illness. As an example In the United States for example, non-adherence to medical regimens has been estimated to cost the US health-care system $100 billion per yr (2-4). Therefore, the outcome of non-adherence is loss: loss of opportunities for patients to improve their health, and loss of medication efficacy by health-care systems, with the subsequent effect of increased morbidity. However, adherence issues in PCOS patients have not been examined thoroughly. Patients report prolonged periods of treatment and side effects of the drug as the most common reason for withdrawal from treatment. To improve the effective management of PCOS patients, it is fundamental to understand facilitating and inhibiting factors to treatment adherence. 

Therefore, the aim of this study was to explore PCOS patients’ views about facilitating and inhibiting factors to adherence.

## Materials and methods

**Study design**

This was a qualitative study using the content analysis method. In the conventional content analysis, coding categories are derived directly from the text data (4). Therefore, we used a similar approach as recommended.

**Participants**

Participants with a confirmed diagnosis of PCOS according to Rotterdam criteria attending the outpatient Gynecology Clinics in Yasuj and Kashan, Iran, were invited to participate in the study. Before their clinic appointment, women participated in a semi-structured interview administered by a trained research assistant in a private setting. Participants were eligible if they met each of the criteria presented in table I. Ten women refused to participate; no attempt was made to determine why those women did not contact the researcher.

**Data collection**

Interviews were arranged to suit participants. Researchers facilitated a calm and familiar place to interview patients. A trained qualitative researcher conducted the interviews. The lead author of the study supervised all data collection to ensure quality control. Participants were encouraged to openly discuss their opinions. No personal information including names or other identifying data was obtained and participants were able to leave the study at any time. 

At the start of each interview, the interviewer introduced herself briefly to talk about the purpose of the study and obtain consent to record the interview. In-depth, semi-structured face-to-face interviews were undertakenexplore the ideas, opinions, and perceptions about facilitating /inhibiting factors of adherence to treatment among Iranian patients with PCOS.

Interviews started with general questions (Tell me about your experiences and perceptions about PCOS, What facilitating or inhibiting factors exist in PCOS care?, what is your opinion about making regular scheduled PCOS care visits when not having health or medication problems) with follow-up questions to explore their answers. To avoid participants feeling that there was right or wrong answers, the emphasis was placed on questions starting with “How” and “What” as well as explorative questions. 

Data were collected until saturation of the data was reached; this occurred when no codes were apparent in the data. Saturation point was reached after analysis with participant 18. The duration of each interview depended on the tolerance and interest of the participant and varied from 20 to 89 min (mean 42 min).

**Ethical considerations**

The Ethics Committee of the Yasuj Medical University approved the study (23/2/893). All participants gave informed consent to publish the details of their interviews. Pseudonyms have been used to protect the women’s anonymity.

**Statistical analysis**

Tapes were transcribed verbatim and reviewed repeatedly to extract key sentences and words of the texts; codes was given to each key word or sentence. Then similar codes were grouped to create categories and compacted to form themes. Data entry into MAXQDA and analysis were undertaken.

To enhance rigour and reliability of the data analysis, other researchers in addition to the primary researcher were involved in peer checking. The main investigator read the transcripts to confirm the coding and categories and then checked the primary researcher’s interpretations. Credibility was enhanced by member checkingby a few participants contacted after the analysis. The overall level of agreement about peer checking and member checking was above 95%. The maximum variation of sampling and prolonged engagement increased the credibility of data.

## Results

This study comprised of semi-structured interviews with 20 patients with PCOS. The characteristics of participants showed maximum variation within the sample. Patients were aged between 21-34 yr; having primary to higher educational level; and had experienced PCOS from 1-10 yr. Five main themes emerged from the analysis: inhibiting factors to treatment adherence which included financial issues, factors related to patients themselves, to the disease, and to the healthcare provider system. Social factors included both facilitating and inhibiting factors (Table II). 

**Financial issues as inhibiting factor to treatment adherence**

In this study, the participants reported the money as an inhibiting factor to adherence. For example: “My main problem is that I can’t visit a good gynecologist for its cost. It is a big burden for me.” participant [P aged 22].

Another patient stated: “I went to gynecologist twice and never followed it up because of financial problems. The drug was very expensive. Now, I got loan to continue the treatment.” [P aged 25].

**Patients themselves as inhibiting factor to treatment adherence**

In-depth interviews with all participants recognized the following patient-related inhibiting factors to adherence with the treatment: 

a) Perception about PCOS

A 27-year-old participant told us about the wrong perception she held about PCOS: “Because I heard that PCOS is something usual in women on television, it was also usual for me. In other word, I thought that I wasn’t alone; there were many people like me. Therefore, I quitted the treatment.” [P aged 27].

b) Knowledge about PCOS 

Some of the participants pointed out that they did not have any information about PCOS which considered as inhibiting factor in following up of the treatment: “I didn't know what PCOS was; I thought that it was a disease that would be resolved after six months. I didn’t think that this disease was ongoing” [P aged 24]. A 25 year old participant told us about wrong knowledge about PCOS: “I initially had menstrual irregularities problem, but since this is usual in my family and all have this problem, I never followed up.” [P aged 25].

**Social factors included both facilitating and inhibiting factors**

a) Family support

Better understanding among family members was something that improved adherence. Some interviewees recalled how their families helped them: “My family always gives me comfort that it’s not a big deal. Even, they come to clinic with me. They always say that it’s nothing, you will be pregnant, we were like you too.” [P aged 22].

One interviewee said: “My husband always reminds me that I used to take my drugs regularly. He kept telling me not to forget my pills. He had supported all around my life.” [P aged 26]. However, some of the participants reported non accompaniment of their husbands as an inhibiting factor of adherence: “When the doctor advised me to have intercourse with my husband for pregnancy every other day, my husband didn’t accept it. He said it was ridiculous. If those plans worked, we would have a baby since four years ago.” [P aged 27].

b) Friends support

Some interviewees recalled how their friends helped them in following up the treatment. One interviewee, a 27-year-old woman, told us about the positive effects of friend support as an inhibiting factor to adherence: “One of my friends comes to our home every time, she tells us promising and hopeful words. My mood improved and I followed my treatment.” [P aged 27].

c) Peer support

Some participants pointed out to positive and others to negative roles of peers in adherence to treatment. For example: “I spoke to those people just like me. They told me that they spent several million Tomans Iranian currencies. It did not work. Do not seek the treatment. Therefore, I did not go for the treatment.” [P aged 27]. A 24-year-old participant talked about peer support as an inhibiting factor to adherence: “For the diagnosis of PCOS, the doctor advised hysterosalpingography. Others told me it was very painful. I feared and did not follow it up.” [P aged 24].

**Disease as inhibiting factor to treatment adherence**

Disease-related issues including side effects and prolonged time required for treatments are the most discussed themes in this study.

a) Side effects of medication

Most participants had experienced side effects of drugs including pain related diagnosis or evaluation of drug consumption, lethargy, anorexia, hot flushing, and mastalgia which increased non-adherence: “When taking these medications, I was always hot with hot flashes. I felt too much pain in my lower abdomen.” [P aged 27]. One interviewee said: “Taking the pills, I was too anorexic, I had very hot flash.” [P aged 24].

b) Prolong time needed for treatment 

The length of the time taken for the entire process was mentioned as an inhibiting factor: stay on the waiting list to visit a doctor in addition to the subsequent time needed for the treatment of PCOS took ‘too long’. “To visit a gynecologist, I had to sit from morning to noon in the waiting room of the clinics. It is extremely frustrating so I didn’t visit the doctor for about 2 months.” [P aged 27]. Another one stated: “I cannot put up with going to doctor repeatedly. For that reason, I discontinued following up the treatment.” [P aged 28].

**Healthcare provider system as inhibiting factor to treatment adherence**

Participants’ experience revealed that health care providers’ negligence to recognize the patient’s psychosocial needs for an effective adherence especially the below issues are considered inhibiting factors to the treatment:

a) Lack of informational support

Not informing the participants about the options of the treatment during the first consultation was considered as inhibiting factor: “My doctor did not explain about my disease at all. She only prescribed my drugs.” [P aged 27]. “Whenever I went to the clinic, she asked me to get ready for sonography. After that, she prescribed drugs and said good-bye! This ambiguous situation is too excruciating for me.” [P aged 34].

b) Absent of holistic care 

Some participants pointed out that they had experienced the inattention to the health care providers about the whole needs for privacy, both emotional and physical, and continued: “Every time, 3-4 patients were also inside the room with the doctor. When I had a vaginal ultrasound, several women were sitting inside the room and it was very inconvenient.” [P aged 26]. “Every time, some patients were inside the room and I was embarrassed that someone would hear my words. Several times, I decided to tell her about my relation with my husband but it’s impossible in the presence of the others.” [P aged 28].

**Table I:** Inclusion criteria

Desire to participate in the study
Being 15-40 ys old
Married
Absence of non-classic adrenal hyperplasia, thyroid dysfunction, hyperprolactinemia
Having two of the following Rotterdam diagnostic criteria: 1) Polycystic ovaries visualized on ultrasound scan (presence of 12 follicles or more in one or both ovaries and/or increased ovarian volume >10 ml) 2) Clinical signs of hyperandrogenism (hirsutism score based on hirsutism score greater than 7 or obvious acne), 3) Having an interval between menstrual periods >35 days and/or amenorrhea, defined as the absence of vaginal bleeding for at least 6 months (i.e. 199 days)
No problems in speaking or listening
Iranian
Not taking any prescription medication (except allergy medications and occasional pain medications) for at least three months before entering the study

**Table II:** Main themes

Financial issues as inhibiting factor to treatment adherence Patients themselves as inhibiting factor to treatment adherence
2a) Perception about PCOS 2b) Knowledge about PCOS
Disease as inhibiting factor to treatment adherence 3a) Side effects of medication 3b) Prolong time needed for treatment
Healthcare provider system as inhibiting factor to treatment adherence 4a) lack of informational support 4b) Absent of holistic care
Social factors included both facilitating and inhibiting factors 5a) Family support 5b) Friend support 5c) Peer support

PCOS: polycystic ovary syndrome

## Discussion

The findings from this qualitative study indicated that there were five reasons for treatment non-adherence among patients with PCOS. In PCOS treatment, when combined as the cause of sub-fertility, successful outcomes of treatment depend upon patient adherence to the prescribed treatment. In our study, failure of the healthcare professional to provide information and lack of dignified care were described as inhibiting factors to treatment adherence. No other studies have found this to be an influencing factor in treatment adherence. Previous studies into adherence to medication suggest factors related to the number of drugs, dosage, treatment period, cost and the time between starting to observe clinical effects rather than the relationship with healthcare professionals (6, 7). 

In addition, most patients in this study expressed concerns about dignity, privacy, respect, and communication. These are in agreement with previous studies where women with PCOS felt poor communication with health care systems and their emotional and psychosocial needs were not met (8-10). Lack of information has been shown to be associated with the poor health-related quality of life in this group (11). Providing information improves the quality of life in women with PCOS and education about their condition improves their understanding of causes and treatment choices (12). Women with PCOS would like to receive information about their conditions from doctors (13). In a study by Weiss et al many of PCOS women in regard to patient-provider relationship reported frustration with delays in diagnosis and distress about the limited information they received about PCOS, and what they needed to truly take control of their health (14). Moreover, in another qualitative study, it was demonstrated that participants described receiving insufficient information from health care professionals and negative experiences in relation to the diagnosis and management of their condition (15). 

However, inattention to the holistic care of patients remains a problem for other conditions (16). The previous study suggested that one reason for non-adherence with health care plan is inattention to psychosocial needs of the patients and only focus on the medical prescription (17). Health care providers played an important role in supporting and encouraging the patients to adhere to their treatments. Good relations with patients improved adherence as it has been found before (18). Health care providers who spent time explaining issues to the patients positively influenced the adherence (19). Care providers should promote optimal adherence by giving clear instructions, providing adequate help to handle possible side effects of the treatment in order to get better adherence. The previous study reported that patient-based care is more required than diseasebased approach with attention to all of the patients’ needs including physical, psychological, social, mental, and spiritual needs. Patient-based care helps the patient to improve the effective engagement to manage their diseases. According to the results of several studies, the interactions between physicians and healthcare team cause to improve the continuity of patient care and disease management (20, 21). Some studies suggested that patients’ education and involvement in the treatment practice can improve health care team efforts for the management of a chronic disease through a set of interactions with the patient and family members (22).

Based on the participants’ views, social support was found to be crucial for patients' treatment. Better understanding among community members was spoken of as something that improved adherence. The previous study has shown that adherence is 1.74 times higher in patients from supportive families (23). The finding of family support as a motivator of PCOS treatment was similar to that of other studies that found those with family support was more likely to practice treatment (24-26). However, in our study negative role of peer support was as inhibiting factors in adherence with treatment that needs more attention in a future study. It is known that families can be supportive by encouraging patients to engage in physical activity (27). Social relationships are an important factor in maintaining changes, and peer support as a facilitator to behavior change is also recognized (28, 29). It seemed that support and assistance from a family member or friend would foster more positive attitudes toward disease.

Moreover, financial status was mentioned as inhibiting factor to the adherence of participants. Insurance status may have prevented patients from following up the treatment. Participants mentioned that they often could not afford to visit the health care providers to repeat prescription schedule. Thus, financial issues emerged as a key theme. Participants were worried and reported that monthly repeated prescriptions caused difficulties in adherence. This is a useful finding and it is important that policy makers working toward the practice of this disease consider subsidy and insurance when designing PCOS treatment programs.

Finally, our findings indicated that patients’ beliefs and knowledge regarding disease strongly influenced their adherence. The previous study associated with antiviral therapy in HIV related treatment has depicted that education levels and educational programs affect adherence (30). It seemed that providing better information about PCOS increases the patient maintenance in the treatment. Health care providers should offer information and consult through cables such as TV, newspapers, and media to increase attention focus on this disease.

Although this study was strong in many ways, it was not without limitations. Simply because this study was intended to be a qualitative study, the main limitation was related to the sample size and number of participants; this, in turn, limits generalization. The study was carried out with women who had sought care in health clinics and it might not be representative of the general population of the women. Moreover, the study was conducted in limited geographical areas and may not reflect the experiences of Iranian women.

## Conclusion

The findings suggest that successful adherence to PCOS treatment is highly dependent on removing a range of different inhibiting factors including financial, social, and health care related inhibiting factors. Indeed it is crucial for clinicians and policy makers to recognize these key inhibiting factors in order to improve treatment outcomes.
